# The “Infernaccio” Gorges: Microbial Diversity of Black Deposits and Isolation of Manganese-Solubilizing Bacteria

**DOI:** 10.3390/biology11081204

**Published:** 2022-08-11

**Authors:** Beatrice Farda, Rihab Djebaili, Maddalena Del Gallo, Claudia Ercole, Fabio Bellatreccia, Marika Pellegrini

**Affiliations:** 1 Department of Life, Health and Environmental Sciences, University of L’Aquila, Via Vetoio, Coppito, 67100 L’Aquila, Italy; 2 Dipartimento di Scienze, Università Roma Tre, Largo San Leonardo Murialdo 1, 00146 Roma, Italy

**Keywords:** geomicrobiology, XRPD, SEM-EDS, 16S rRNA gene metabarcoding, Mn-oxide-solubilizing bacteria

## Abstract

**Simple Summary:**

“Infernaccio” gorges are one of the Earth’s hidden habitats in Central Italy. Beyond the deep incisions and high slopes, these gorges are characterized by black deposits in gorge walls and covering rock surfaces. Several geological events have shaped these unique geological formations and their microbiota. This study investigated microbial contribution to black deposit formation and isolating Mn-oxide-solubilizing bacteria. Our results provided evidence of the putative role of Bacteria and Archaea in forming manganese oxide deposits. Findings also showed that these deposits are a source of valuable strains with manganese oxide bioleaching properties, essential for bioremediation and metal recovery.

**Abstract:**

The present study explored the microbial diversity of black deposits found in the “Infernaccio” gorge. X-ray Powdered Diffraction (XRPD) was used to investigate the crystallinity of the samples and to identify the minerals. Scanning electron microscope and energy-dispersive X-ray spectroscopy (SEM-EDS) were used to detect the bacterial imprints, analyze microbe–mineral interactions, and highlight the chemical element distribution in the black deposits. 16S rRNA gene metabarcoding allowed the study of Archaea and Bacteria communities. Mn-oxide-solubilizing isolates were also obtained and characterized by culturable and molecular approaches. The multidisciplinary approach showed the occurrence of deposits composed of birnessite, diopside, halloysite, and leucite. Numerous bacterial imprints confirmed the role of microorganisms in forming these deposits. The Bacteria and Archaea communities associated with these deposits and runoff waters are dynamic and shaped by seasonal changes. The uncultured and unknown taxa are the most common and abundant. These amplicon sequence variants (ASVs) were mainly assigned to Proteobacteria and Bacteroidetes phyla. Six isolates showed interesting Mn solubilization abilities under microaerophilic conditions. Molecular characterization associated isolates to *Brevibacterium*, *Bacillus*, *Neobacillus*, and *Rhodococcus* genera. The findings enriched our knowledge of geomicrobiological aspects of one of the Earth’s hidden habitats. The study also unveiled the potential of this environment as an isolation source of biotechnologically relevant bacteria.

## 1. Introduction

Manganese (Mn) is a widespread transition metal on the Earth’s surface (about 0.1%), found in varying concentrations in different geochemical spheres. Manganese can exist in seven oxidation states (0 to +7), but the chemical species commonly found in natural environments are Mn(II), Mn(III), and Mn(IV) [1,2]. Mn valence states depend mainly on the pH and the environmental system’s redox potential (Eh) [3]. The reduced form, Mn(II), is generally soluble and stable in the absence of oxygen; the more oxidized form, Mn(IV), is insoluble and tends to form oxides, often in the form of deposits.

Microorganisms are primarily implicated in Mn transformation and sedimentation in terrestrial and aquatic environments [4]. For example, Polgári et al. investigated microbial activity in Jurassic Mn-carbonate ore formation (Úrkút, Hungary). Micro-laminae revealed a depositional series of microbial mats that produced sedimentary structures. These structures suggested a microbial growth daily rhythmicity and a biological formation of manganese ores [5]. The list of microorganisms implicated in Mn oxidations and reductions is continuously growing. Among the known microbes involved in transformations of Mn are conventional bacteria, such as *Arthrobacter*, *Pseudomonas*, and *Bacillus*, prosthecate bacteria (*Pedomicrobium*, *Hyphomicrobium*, *Metallogenium*), encapsulated bacteria (e.g., *Leptothrix discophora*), and fungi (*Cephalosporium*, *Cladosporium*, *Aspergillus*) [6]. Several mechanisms have been proposed for the microbial transformation of Mn from one oxidation state to another. If the effects involve changes in the microenvironment (e.g., Eh and pH modification, adsorption of preformed oxides on the cell surface, and the presence of organic complexes) around the microbial surface, the microbial contribution is indirect. For instance, Biondi and collaborators explained the genesis of Urucum Mn ores (Brazil) through complex diagenetic processes. They include the decomposition and mineralization of cellular and extracellular polymeric substances from iron and manganese bacteria [7]. The microbial involvement is direct if the transformations result from enzymatic oxidations or reductions. Depending on the ecosystem and environmental conditions, many enzymatic mechanisms can be involved in oxidation and reduction reactions [4].

Microbial oxidation of Mn(II) has been extensively studied by molecular biology techniques on three model bacteria: *L. discophora*, *Bacillus* sp. strain SG-1, and *Pseudomonas putida* strains MnB1 and GB-1 [8]. These microorganisms possess Mn(II) oxidation genes and share gene sequences similar to MultiCopper oxidase (MCOs) enzymes. These organisms and *Pedomicrobium* sp. and *Erythrobacter* SD-21 showed biochemical evidence consistent with the involvement of copper enzymes in Mn(II) oxidation. Other classes of Mn(II)-oxidizing bacteria use proteins similar to animal Heme-dependent peroxidases (AHPs) (e.g., myeloperoxidases and lactoperoxidases). This enzyme class uses hydrogen peroxide (H_2_O_2_) as the final electron acceptor and contains a heme group that functions as a cofactor [9]. This type of enzyme has been identified in *Erythrobacter* sp. strain SD21 and *Aurantimonas manganoxydans* strain SI85-9A1. Caspi et al. proposed the first evidence of genes for the RubisCO enzyme in strain SI85-9A1, a marine α-proteobacterium that oxidizes the Mn from Mn(II) to Mn(IV). This strain has genes like cbbL, the gene encoding for the large subunit of the ribulose-1,5-bisphosphate carboxylase/oxidase (RubisCO) enzyme [10].

Mn microbial solubilization and the role of Mn-reducing microorganisms in different ecosystems have attracted many scientists’ attention for decades. Nevertheless, investigating the enzymatic mechanisms behind these transformations has always been very complex due to the heterogeneity of environmental conditions and naturally occurring manganese oxides. Many authors agree that the transformation of Mn(IV) to Mn(II) is a process carried out by microorganisms using both endogenous and exogenous substrates as electron acceptors (e.g., lactate and acetate) [11,12]. It has also been observed that oxygen is necessary for cultures to adapt to Mn(IV) reduction. The reduction rate does not change significantly under anaerobic conditions [13].

Microbiological processes contribute significantly to biogeochemical cycling and can be exploited biotechnologically to recover metals [14]. Biomining is a broad term that encompasses bioleaching, bio-oxidation, and biomineralization processes [15]. Among them, the bioleaching mechanism of Mn is primarily indirect, involving the generation of organic acids in the leaching media, which reduces Mn oxides [16]. Bioleaching technologies for metal extraction have enormous potential. They will play a crucial part in the rapidly evolving field of raw supplies, ever-decreasing mineral levels, geographically scattered mineralization forms, and lack of universal applicability in physical processing [17].

We hypothesized the role of microorganisms in “Infernaccio” gorge (Viterbo, Italy) Mn deposit formations and that these oxides could be a valuable source of microorganisms helpful in Mn bioleaching. To test these hypotheses, we studied the deposits found in gorge sites with a multidisciplinary approach. The mineral composition of the samples was studied by X-ray Powdered Diffraction (XRPD). To reveal the presence of bacteria imprints and element distribution within the samples, scanning electron microscopy and microanalyses (SEM-EDS) were used. Cultural and molecular approaches were employed to study microbial diversity. The cultural approach was directed toward the microaerophilic flora responsible for Mn solubilization. The molecular approach was directed toward the study of the Archaea and Bacteria microbial community, sequencing the V3–V4 variable region of the 16S rRNA gene.

## 2. Materials and Methods

### 2.1. Geological Description of the Sampling Area

The sampling site at the “Infernaccio” gorge, located in the Viterbo province (Lazio, Italy—42°31′40.85″ N; 12°7′48.24″ E), is part of the “Tuscia” area. Gorges are a unique and distinguishing feature due to their extensive and branching hydrographic network. The activity of three major volcanic complexes (Vulsino, Vicano, and Cimino) formed a pyroclastic layer (lava and ignimbrite deposits). The erosive action of waters resulted in gorges’ deep incisions.

### 2.2. Sampling and Sample Handling

**Figure 1:**
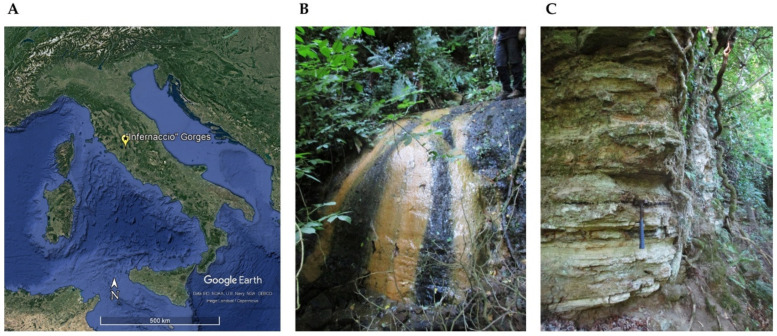
Location map ((**A**), obtained from Google Earth Pro) and sampling sites of sample S1 and S2 ((**B**), black crust on stream rocks and runoff water, respectively) and S3 ((**C**), black crust within a gorge wall).

Samplings were carried out on three different points of the Gorge—black crust on stream rocks (S1), runoff water (S2), and black crust within a gorge wall (S3)—during February (I) and July 2020 (II) (Figure 1). Samples S1 and S3 were taken with a geologist’s hammer and collected in sterile tubes. Sample S2 was picked with sterile tubes. All specimens were kept under refrigeration and transferred to the laboratory. Three aliquots of each sample were independently processed immediately for the cultural approach, as described in paragraph 2.6. For mineralogical investigations, samples were stored in the fridge (+4 °C). For molecular analyses, five aliquots of each sample were chosen, pooled, and put into an RNAlater solution (Ambion, Austin, TX, USA) according to the manufacturer’s instructions. All the samples were kept at −80 °C until they were tested.

### 2.3. DNA Extraction and 16S rRNA Gene Metabarcoding

Genomic DNA extraction was performed using 500 mg of powdered samples and bead-beating methods according to the NucleoSpin^®^ Soil kit (Macherey Nagel, Düren, Germany) manufacturer’s instructions. Each sample was extracted thrice. DNA concentration and purity were checked by a Nanodrop spectrophotometer (Thermo Scientific^TM^, Waltham, MA, USA) and a Qubit fluorometer (Thermo Scientific^TM^). For each sample, the replicates were combined in an equimolar combination. Using the analytical approach previously outlined [6], we focused the analysis on the V3 and V4 regions of the 16S rRNA gene [18], using paired-end 16S rRNA gene community sequencing on the Mi-Seq Illumina platform (Bio-Fab Research, Rome, Italy). After filtering, the readings were examined for quality and counted. The DADA2 plugin was used to assemble the ASVs (Amplicon Sequence Variants) using QIIME2 (qiime2-2020.2 version) [19]. The V3–V4 specific area was extracted from the 16S file retrieved from the SILVA 138 database (https://www.arb-silva.de/ accessed on 28 October 2021) and utilized for classifier training via the fit-classifier-naive-bayes plugin. For the taxonomic assignment, a 97% similitude was used.

### 2.4. Mineralogical Investigation by XRPD

The natural mineral samples were analyzed as previously described [20] by the X-Ray Powder Diffraction (XRPD) technique at “Laboratorio di Diffrazione ai Raggi X” (Department of Science, University of Roma Tre). Analyses were performed by a Scintag X1 diffractometer with fixed divergence slits and a Peltier-cooled Si(Li) detector (resolution < 200 eV) under CuK1 radiation (λ 1.54055, 40 mÅ, 45 kV). The incoming beam had a diverging slit width of 2 mm and a scatter-slit width of 4 mm; the diffracted beam had a receiving slit width of 0.5 mm and a scatter-slit width of 0.2 mm. The acquisitions were performed in step-scan mode (step size of 0.05° 2θ; counting duration of 3 s/step in the 5–70° 2θ range).

### 2.5. Sample Characterization by SEM-EDS

The morphological and geochemical features of samples were examined using scanning electron microscopy (SEM) and energy-dispersive X-ray spectroscopy (EDS). A Zeiss Gemini500 scanning electron microscope was used to analyze the samples, and an INCA X-ACT PELTIER COOLED detector was used to perform EDS Microanalysis (Aztec Energy, Oxford). For SEM acquisitions, different Electron High Tension (EHT) and Work Distance (WD) parameters were used (details provided in figure captions).

### 2.6. Isolation and Characterization of Culturable Solubilizing Bacteria

Mn-oxide-solubilizing bacteria were isolated in manganese basal medium (MMB) containing MnO_2_. [11]. Bacterial suspensions of the samples (obtained by stirring, for 30 min, 1 g of powdered sample with 9 mL of 1% tween 20 saline solution) were centrifuged at 3000 RPM for 10 min, and 100 µL of the serial dilutions (10^−2^, 10^−3^, 10^−4^) of supernatant were plated on MMB. Plates were incubated microaerophilically at 30 °C until the complete development of visible colonies in the plates (20 days) in anaerobic jars and using anaerobic generation kits (Oxoid, Basingstoke, UK). Different colonies were identified and purified on MMB using the smear technique. Colonies with high Mn-oxide-solubilizing abilities (estimated by MMB discoloration) were further characterized. Bacteriological traits were investigated by qualitative laboratory methodologies (employing reagents and colonies coloration) [21].

### 2.7. 16s Barcoding and Phylogenetic Analysis

The most interesting Mn-oxide-solubilizing strains were also subjected to molecular characterization by 16S barcoding (Microbion, Verona, Italy). Bacterial cultures were subjected to DNA extraction (enzymatic lysis and alcoholic precipitation). DNA was amplified by universal bacterial primers (8F/1541R). Forward and reverse sequences were merged, and consensus sequences (~1400 bp) were compared to those available in the NCBI (National Center for Biotechnology Information; http://www.ncbi.nlm.nih.gov/; accessed 3 May 2022) genetic database. The local base alignment search (BLAST) and sequence identity value greater than 99% were used. The Maximum Likelihood method was used to infer the tree. *Escherichia coli* BL21 was used as an outgroup, and Maximum Likelihood bootstrap analysis repeating the data matrix 1000 times (1000 bootstrap) was performed with RAxML [22], using MegAlign Pro 17 (DNASTAR, Lasergene, Madison, WI, USA).

### 2.8. Statistical Analysis

To create the heatmap of ASVs of main genera, calculate alpha-diversity metrics (i.e., Simpson, Shannon, and Chao1 indices), and generate the taxonomy bar plots of ASVs at Phylum, Class, and Genus levels, Primer 7 and PAST 4.03 software were used. Venn diagrams were created using the Bioinformatics & Evolutionary Genomics program (https://bioinformatics.psb.ugent.be/webtools/Venn/, accessed on 1 March 2022) to highlight the common ASVs among the samples.

## 3. Results

### 3.1. DNA Extraction and 16S rRNA Gene Metabarcoding

To unveil differences among the deposits and evaluate the seasonal changes, samples of the first (S1 I, S2 I, S3 I) and second (S1 II, S2 II, S3 II) sampling campaigns were subjected to DNA extraction and 16S rRNA gene metabarcoding. Results were processed for richness and diversity evaluations (Table 1 and Figure S1). All samples showed very high diversity (Shannon H’ values higher than 3.5). S1 and S2 of the first sampling campaign (S1 I and S2 I) showed a lower number of taxa than the second sampling ones (S1 II and S2 II). S2 I showed more individuals but lower diversity indices than S2 II. For sample S3, similar indices were obtained for the two sampling campaigns. Table 1 also presents the results of relative abundances at the domain level. Archaea were present with abundances lower than 0.5% in all the samples and absent in sample S1 I. The second sampling campaign had higher Archaea abundances than the first one.

**Figure 2:**
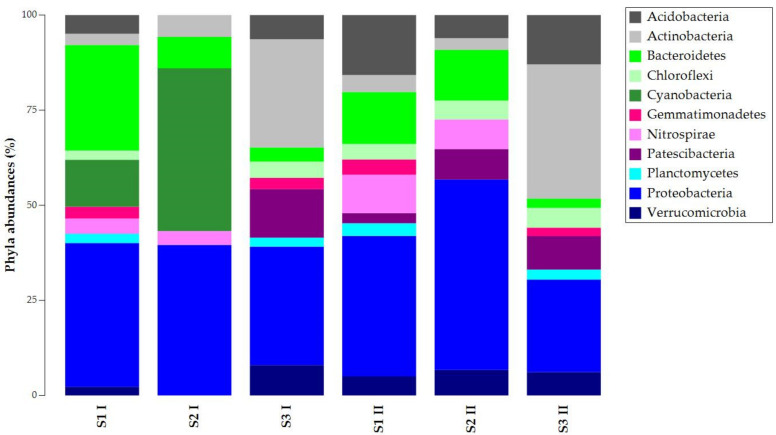
Taxonomy bar plot of ASVs (Amplicon Sequence Variants) at the phylum level.

Results were filtered (2% cutoff) and depicted with taxonomy bar plots to study the composition of ASVs at different taxa levels (phylum, family, genus). Figure 2 shows the ASVs abundances at the phylum level. Proteobacteria was common and abundant in all the samples, while Bacteroidetes presented different abundances per sample. The other phyla had different percentages in the total abundances of the samples. Except for sample S2 I, Acidobacteria, Chloroflexi, and Verrucomicrobia were present in all the samples. S1 I and S2 I were the only samples where Cyanobacteria were found, but they lacked Gemmatonomadetes and Patescibacteria. Nitrospirae was absent in S3 samples, while Planctomycetes was absent in S2 samples.

**Figure 3:**
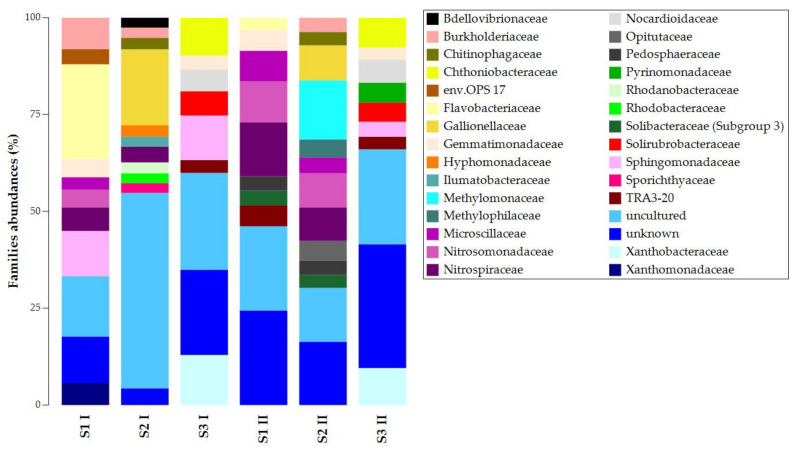
Taxonomy bar plot of ASVs at the family level.

At the family level, we found similarities in unknown and uncultured ASVs (Figure 3). The presence and abundances of the other ASVs showed high dissimilarities among the samples of the same sampling campaign and significant temporal changes (Figure S2). For the first sampling campaign, S1 I and S2 I shared the presence of Nitrospiraceae and Burkholderiaceae, while S1 I and S3 I shared Sphingomonadaceae and Gemmatimonadaceae. For the second sampling campaign, S1 II and S2 II shared more ASVs: Pedosphaeraceae, Solibacteraceae (Subgroup 3), Microscillaceae, Nitrospiraceae, and Nitrosomonadaceae. S1 II and S3 II shared TRA3-20 and Gemmatimonadaceae. Despite the temporal changes’ effects on taxa composition and abundances, S3 presented Chthoniobacteraceae, Nocardioidaceae, Solirubrobacteraceae, and Xanthobacteraceae. Chitinophagaceae and Gallionellaceae were always present in S2. In contrast, Flavobacteriaceae was the only common lineage in S1 I and S1 II.

**Figure 4:**
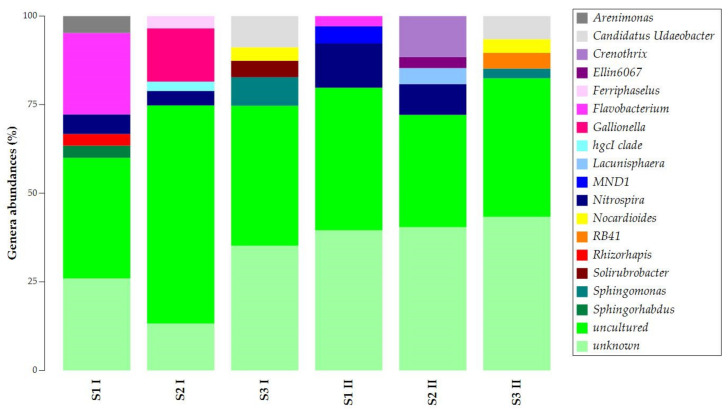
Taxonomy bar plot of ASVs at the genus level.

The uncultured and unknown ASVs at the genus level prevailed in the samples’ abundances, with percentages over 50% (Figure 4). Still, samples obtained in the two seasons presented similarities. *Flavobacterium* was common in S1 I and S1 II. Candidatus *Udaeobacter*, *Nocardioides*, and *Sphingomonas* were common in S3 I and S3 II. For S1 and S2 of both sampling campaigns, *Nitrospira* was found to be common.

The common presence of *Nitrospira* in samples S1 and S2 and the exclusive presence of Candidatus *Udaeobacter*, *Nocardioides*, and *Sphingomonas* in sample S3 allowed the separation of the samples into two main clusters (Figure S3). The first cluster was observed for samples S3 I and S3 II, while the second was for all S1 and S2 samples.

### 3.2. Isolation and Characterization of Culturable Solubilizing Bacteria

Twelve strains were isolated from the three samples on MMB medium. Isolates C17a, C17b, C20a, C20b, C20c, and C22 were isolated from S1; C1b, C14, and C19 from S2; and C16, C21, and C23 from S3. Once purified, the strains’ ability to solubilize Mn was investigated by evaluating the discoloration intensity of MMB. Results on solubilization ability are shown in Table 2.

Based on the Mn solubilization results, six strains were characterized for bacteriological and biochemical traits (Figure S4). The colonies’ morphology and biochemical–bacteriological characteristics are shown in Table 3 and Table 4, respectively. The colonies presented diverse morphology and consistency (Figure S5). All the strains were opaque, and most presented a creamy texture (mucosal for C14). Colonies presented variable sizes and colors. C1b, C14, and C16 were circular and presented regular margins, and smooth surfaces. The other strains showed irregular forms and had undulating margins and rough surfaces.

The bacteriological traits showed that all isolates were Gram-positive. Most strains were rod-shaped, except for C1b, which had coccus morphology. All isolates were spore producers except for strains C1b and C14.

### 3.3. 16s Barcoding and Phylogenetic Analysis

**Figure 5:**
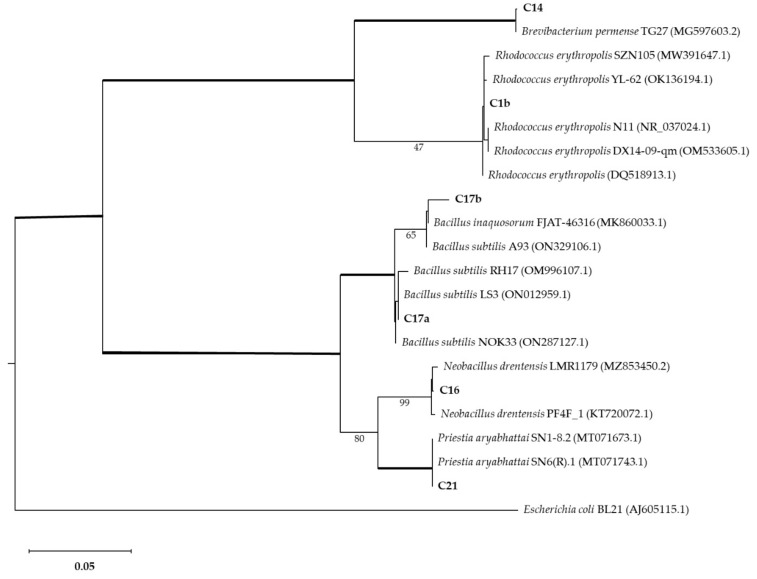
Phylogenetic analysis of bacterial isolates compared with other isolates (GenBank accession numbers shown in brackets). The Maximum Likelihood method was used with a bootstrap consensus tree (from 1000 replicates to represent the distance). *Escherichia coli* BL21 was introduced as an outgroup. Number of substitutions per nucleotide site for a unit of branch length is given in the scale bar (0.05).

The strains with interesting Mn-oxide-solubilizing abilities were further characterized by 16s barcoding. The phylogenetic analysis associated isolates with *Brevibacterium*, *Bacillus*, *Neobacillus*, and *Rhodococcus* genera (Figure 5). Isolate C1b was identified as *Rhodococcus erytropolis* (100% identity). Isolate C14 was identified as *Brevibacterium sediminis* (100% identity). Isolate C16 was identified as *Neobacillus drentensis* (100% identity). Isolates C17a and C17b were identified as *Bacillus subtilis* (100% and 98.7% identity, respectively). Isolate C21 was identified as *Priestia aryabhattai* (100% identity).

### 3.4. Mineralogical Investigation by XRPD

**Figure 6:**
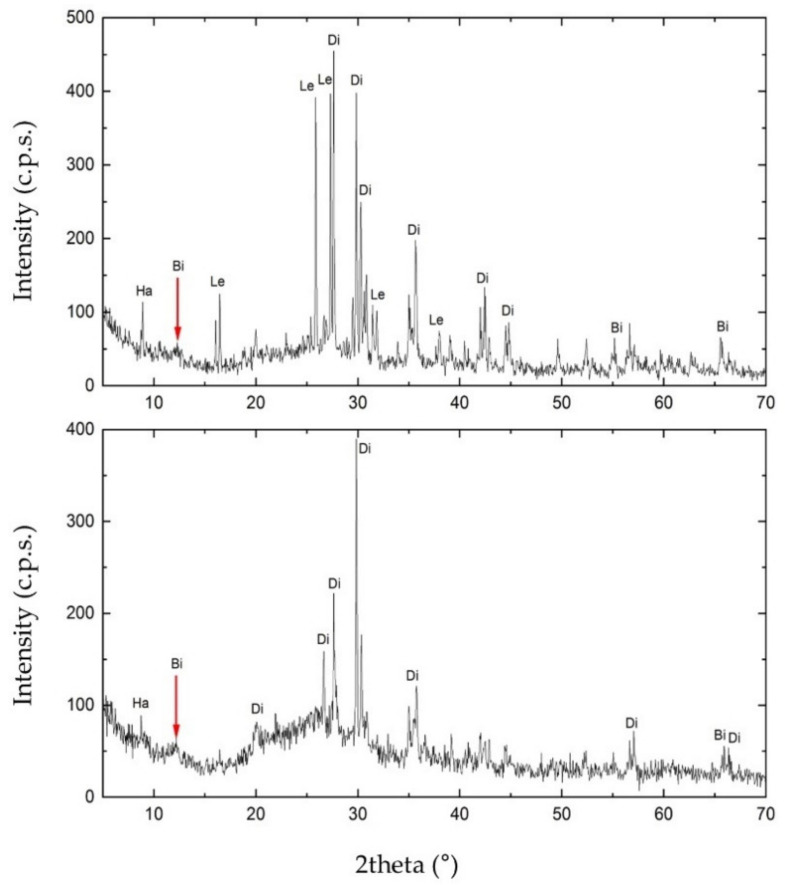
X-ray Powder Diffraction (XRPD) diffractogram obtained for sample S1 (on the top) and S3 (on the bottom). In the figure: Ha, halloysite; Di, diopside; Bi, birnessite; Le, leucite.

To investigate the mineral composition of S1 and S3, we carried out the XRPD analysis. As shown in Figure 6, the matrix rock of both samples was composed of birnessite ((Na,Ca)_0.5_(Mn^4+^,Mn^3+^)2O_4_·1.5H_2_O). Diffractograms also revealed the presence of two silicates in the matrix rock, identified as diopside (CaMgSi2O_6_) and halloysite (Al_2_(Si_2_O_5_)(OH)_4_). S1 also showed the presence of leucite (KAlSi_2_O_6_). Furthermore, in the diffractogram of S3, the increasing trend between 15° and 35° of 2theta may highlight either the presence of one or more amorphous phases. These are attributable to the glassy component of the matrix rock or a contribution of Mn-Fe phases of low crystallinity.

### 3.5. Sample Characterization by SEM-EDS

**Figure 7:**
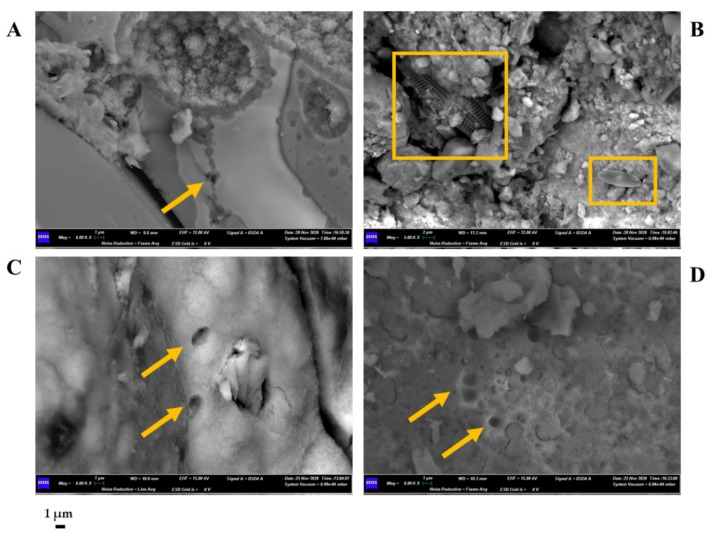
Scanning Electron Microscope (SEM) micrographs obtained for sample S1 (**A**,**B**) and S3 (**C**,**D**). Microbial imprints are highlighted with arrows. Squares indicate the presence of diatoms. Scale bar (1 µm).

**Figure 8:**
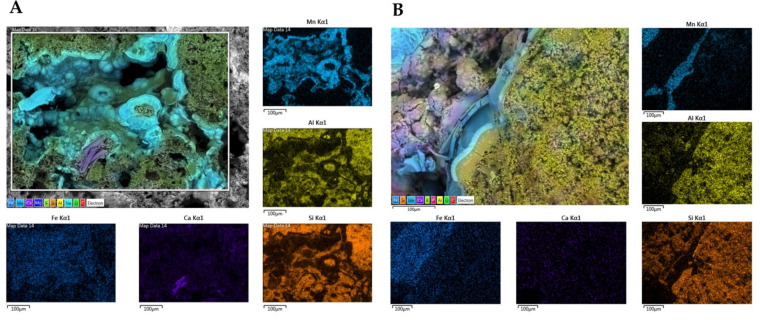
Energy Dispersive X-ray Spectroscopy (EDS) maps of main elements obtained for samples S1 (**A**) and S3 (**B**). The distribution of the single elements (Fe, Ca, Si, Al, and Mn) are provided in subfigures. Scale bars (100 µm).

We performed SEM-EDS analyses of S1 and S3 to investigate bacterial traces and element distribution. They showed the presence of microbial imprints (Figure 7A, C and D), as detected by a cell-like shape of reduced dimensions (1 µm) and distinctive fossil diatoms (Figure 7B). SEM-EDS maps (Figure 8) revealed the presence of chemical elements, such as Mn, Al, Si, Ca, and Fe. Mn is mainly present in the black layers alternating irregularly with interstices. These interstices are filled with silicate materials, as demonstrated by the occurrence of Al and Si elements. This chemical characterization confirmed the presence of aluminosilicates (halloysite and leucite).

Furthermore, Ca seemed to have a more homogeneous and lower occurrence in S4, while it appeared higher in S1 with the evident presence of some precipitates. Their formation could be attributed to the co-precipitation of calcium carbonate (possibly in the calcite form) during the deposition of Mn oxides. Finally, Fe exhibited a relatively homogeneous distribution in both rock samples.

## 4. Discussion

Studying Earth’s unexplored geological formations allows us to enrich our knowledge of interesting biological processes. In this study, we focused on the microbial contribution to the formation of mineral deposits using a multidisciplinary approach. The 16s rRNA gene sequencing revealed dynamic microbial communities with diverse taxa composition and abundances based on the sample type and seasonal changes. Uncultured and unknown bacteria were the ASVs that most accounted for the total abundances, underlining how little we still know about these environmental systems. The presence of common genera in samples S1 (black crusts of rock surfaces) and S2 (runoff waters) allowed us to speculate about the contribution of runoff to black deposits. Microbial communities’ existence and functioning strictly depend on environmental surroundings [23]. In our case, seasonal changes modify runoff water quantity and content. These shifts induce changes in the microbial communities of the waters and rocks subjected to their run-off.

Conversely, the S3 samples (included in the walls) are less subjected to abiotic changes and present low seasonal variations. The strict association between S1 and S2 is also supported by the common presence of *Nitrospira* (common in S1 and S2), a nitrite-oxidizing chemolithoautotrophic genus commonly found in freshwaters. Among the other most relevant common genera, *Flavobacterium* (present in S1 samples) plays a crucial role in Mn removal and oxidation [24,25]. *Nocardioides* and *Sphingomonas* genera (common in S3 I and S3 II) comprise different Mn-oxidizing bacteria [26,27].

XRPD highlighted the presence of birnessite, diopside, leucite, and halloysite. Birnessite is a widespread phase in these geological formations [28]. Diopside and leucite are typical minerals of volcanic rocks, while halloysite is a product of alteration of the glass fraction of the same volcanic rock [29,30]. The sampling site, located in the Viterbo area, is situated within the “Tuscia laziale”, which developed for the most part on volcanic substrates deriving from the explosive activity of three volcanic complexes: Vulsino, Vicano, and Cimino [31]. Between these small volcanoes, low and monotonous tuffaceous plateaus developed, furrowed by deep valleys. The volcanic soils developed on older grounds of sedimentary origin, which can outcrop or emerge from the volcanic cover in a relatively minor way. Less than 2 million years ago, the waters of the Pliocene Sea covered all this emerged area, lapping the Apennine chain [31]. The territory was modified during the Pleistocene. The simultaneous marine regression and the genesis of the three volcanic complexes led to the territory being covered by lava and ignimbrite deposits, subsequently subject to degradation [32].

This geological history characterizing the sampling site also explains the presence of numerous fossils/traces of diatoms (unicellular eukaryotic “brown” algae living in aquatic environments) highlighted with SEM observations. SEM-EDS observations and microanalysis showed that the inclusions within the Mn samples did not have a homogeneous morphology determined by overlapping and oriented layers. The concretions and amorphous deposits suggest a putative microbial role in enhancing the Mn(II) oxidation rate and in the subsequent deposition phase of Mn oxides [6,20]. This amorphous manganese oxide shape was also found in ferromanganese deposits linked to Neyriz ophiolite-colored mélange in the Abadeh-Tashk area (Fars Province, Iran). Filamentous beads highlighted the implication of microorganisms in Fe and Mn precipitation. The latter had regular circular forms, vermiform structures, depositional series of biomats, traces of embedded organic material, and REE concentrations in Mn ores [33].

Moreover, the identification of “biosignatures” through SEM observations underlined the involvement of bacteria in the formation of the Mn deposits. Microbial fossils and imprints are common in the laminated Mn-carbonate ores, indicating a putative role of microorganisms in the deposition and mineralization of ores. For example, Yu et al. studied Datangpo Formation Mn deposits in South China, and they inferred that microbial enzyme activity formed crystallized Mn oxide/hydroxides and carbonaceous material via diagenesis [34]. Cellular imprints can be traced back to the pre-divisional stage of prostrate dimorphic bacteria, such as *Pedomicrobium* sp. and *Hyphomicrobium* sp., as previously reported for other Mn crusts of caves [6].

The “Infernaccio” gorges are a valuable source for Mn-oxide-solubilizing microorganisms. Isolates showed both aerobic and anaerobic metabolisms (induced by microaerophilic conditions). Spore-producing abilities were also described. The latter is a helpful trait for overcoming stress and exploiting strains for biotechnological applications. Among the Mn-oxide-solubilizing bacteria, most of the isolates belonged to *Bacillus* spp. Several species of this genus have been described for their Mn solubilization abilities under aerobic and anaerobic conditions [35]. Sanket et al. described Mn solubilization by the strains *B. cereus* AMSB3 and *B. nealsonii* AMSB4 isolated from mine water samples [36]. Kanso et al. isolated a manganese-reducing *B. subterraneus* strain from a thermal aquifer [37]. Sujith et al. described Mn-reducing abilities for *Brevibacterium epidermidis* NA5 [38]. *P. aryabhattai* is a novel Bacillaceae genus [39], previously known as *Bacillus aryabhattai*, which has also been described for Mn-oxide-solubilizing abilities [35]. *Brevibacterium* spp. actively transport Mn [40], and their ribonucleotide synthesis is strictly linked to the presence of this element [41]. No previous reports on the Mn solubilization abilities for *Neobacillus* strains are reported in the literature, while manganese-resistant *R. erythropolis* strains were isolated from deep-sea nodules [42,43,44,45,46]. Even if mostly belonging to already known Mn-oxide-solubilizing genera, the present study described new species involved in Mn transformations. Enriching the knowledge of this group of bacteria is relevant to environments. The increasing demand for Mn and the continuous overexploitation of natural resources lead to a depletion of mineral reserves and increased environmental pollution [47]. Mn is widely used in industrial mass production (such as steel, pipes, batteries, glass and ceramic objects, dyes, and medicines) and is the fourth most used metal after iron, aluminum, and copper [48]. These minerals are extracted through invasive processes, such as acid drainage or the creation of massive mines. Waste products are generated during these processes, leading to environmental pollution (air, soil, and water) and severe risks to humans [49].

## 5. Conclusions

The present study investigated the “Infernaccio” gorge to unveil the microbial contribution to their formation and to evaluate the possibility of using them as an isolation source of Mn-oxide-solubilizing bacteria. The multidisciplinary approach confirmed the microbial role in forming these Mn oxides. Six isolates showed good Mn solubilization abilities under microaerophilic conditions. Molecular identification associated isolates with *Brevibacterium*, *Bacillus, Neobacillus,* and *Rhodococcus* genera. The description of these abilities in species that have never been reported before opens further investigations into the Mn-oxide-solubilizing mechanisms and their involvement in the cycling of other minerals. 

Further studies should investigate the potentialities for the technological application of selected strains in the bioleaching of Mn. The biochemical (e.g., enzymes) and metabolic (e.g., carbon sources utilization) traits should be further investigated by standardized quantitative methods. However, the investigated traits (especially for *Bacillus* spp.) show great potential in Mn biomining. More in-depth studies are needed to describe these deposits’ morpho-mineralogical and geochemical nature adequately. Nevertheless, our findings enriched the knowledge on the Mn deposits of gorges, which were previously poorly investigated, and we described the “Infernaccio” gorges for the first time. These findings could be relevant for understanding the origin of these deposits and remarking on the importance of protecting these ecosystems, usually subjected to human tourism.

## Figures and Tables

**Table 1:** Diversity indices calculated on 16s rRNA gene metabarcoding results through PAST 4.03 and abundances (%) at the domain level.

	S1 I	S2 I	S3 I	S1 II	S2 II	S3 II
Taxa S	374	358	858	872	633	827
Individuals	4860	12439	16651	11483	9702	15253
Simpson 1-D	0.992	0.861	0.995	0.997	0.995	0.997
Shannon H’	5.50	3.76	6.07	6.39	5.92	6.25
Chao-1	374	358	858	872.4	633.1	827
**Domain-level abundances**						
Archaea	0.0	0.1	0.2	0.3	0.2	0.5
Bacteria	100.0	99.9	99.8	99.7	99.8	99.5

**Table 2:** Mn solubilization abilities by visual discoloration of manganese minimal basal (MMB) medium of the twelve isolates. The solubilization abilities were indicated as follows: high discoloration (+++); medium discoloration (++); low discoloration (+); no discoloration (−).

	C1b	C14	C16	C17a	C17b	C19	C20a	C20b	C20c	C21	C22	C23
MMB Discoloration Rate	+	+	+++	++	++	−	−	−	−	++	−	−

**Table 3:** Morphological characteristics and Gram staining of the isolates with Mn-oxide-solubilizing abilities.

	C1b	C14	C16	C17a	C17b	C21
Size	Large	Large	Small	Medium	Medium	Medium
Color	Pink	Clear pink	White	Clear brown	Clear brown	Clear brown
Form	Circular	Circular	Circular	Irregular	Irregular	Irregular
Margin	Regular	Regular	Regular	Undulating	Undulating	Undulating
Surface	Smooth	Smooth	Smooth	Rough	Rough	Rough
Transparence	Opaque	Opaque	Opaque	Opaque	Opaque	Opaque
Texture	Creamy	Mucosal	Creamy	Creamy	Creamy	Creamy

**Table 4:** Characteristics of the isolates with Mn-oxide-solubilizing abilities.

	C1b	C14	C16	C17a	C17b	C21
Spores	no	no	yes	yes	yes	yes
Gram reaction	+	+	+	+	+	+
Morphology	coccus	rod	rod	rod	rod	rod

## Data Availability

The datasets generated and/or analyzed during the current study are available from the corresponding author on reasonable request.

## Supplementary Materials

The following supporting information can be downloaded at: https://www.mdpi.com/article/10.3390/biology11081204/s1. Figure S1. Species accumulation plot showing the number of amplicon sequence variants (ASVs) on sample size for each sample. Figure S2. Venn diagram underlining the common ASVs (given in brackets) at the family level. Figure S3. Heatmap of the main genera found in the samples. Figure S4. Summary of the experiment setup during Mn-oxide-solubilizing process. Figure S5. Mn-oxide-solubilizing isolates on MMB medium.

## References

[1] Geszvain K., Butterfield C., Davis R.E., Madison A.S., Lee S.-W., Parker D.L., Soldatova A., Spiro T.G., Luther G.W., Tebo B.M. (2012). The Molecular Biogeochemistry of Manganese(II) Oxidation. Biochem. Soc. Trans..

[2] Marshall K.C. (1979). Chapter 5 Biogeochemistry of Manganese Minerals. Studies in Environmental Science.

[3] Gad S.C. (2005). Manganese. Encyclopedia of Toxicology.

[4] Wang X., Xie G.-J., Tian N., Dang C.-C., Cai C., Ding J., Liu B.-F., Xing D.-F., Ren N.-Q. (2022). Anaerobic Microbial Manganese Oxidation and Reduction: A Critical Review. Sci. Total Environ..

[5] Polgari M., Hein J.R., Toth A.L., Pal-Molnár E., Vigh T., Biró L., Fintor K. (2012). Microbial Action Formed Jurassic Mn-Carbonate Ore Deposit in Only a Few Hundred Years (Úrkút, Hungary). Geology.

[6] Vaccarelli I., Matteucci F., Pellegrini M., Bellatreccia F., del Gallo M. (2021). Exploring Microbial Biosignatures in Mn-Deposits of Deep Biosphere: A Preliminary Cross-Disciplinary Approach to Investigate Geomicrobiological Interactions in a Cave in Central Italy. Front. Earth Sci..

[7] Biondi J.C., Polgári M., Gyollai I., Fintor K., Kovács I., Fekete J., Mojzsis S.J. (2020). Biogenesis of the Neoproterozoic Kremydilite Manganese Ores from Urucum (Brazil)—A New Manganese Ore Type. Precambrian Res..

[8] Tebo B.M., Bargar J.R., Clement B.G., Dick G.J., Murray K.J., Parker D., Verity R., Webb S.M. (2004). Biogenic Manganese Oxides: Properties and Mechanisms of Formation. Annu. Rev. Earth Planet. Sci..

[9] Barboza N.R., Guerra-Sá R., Leão V.A. (2016). Mechanisms of Manganese Bioremediation by Microbes: An Overview. J. Chem. Technol. Biotechnol..

[10] Caspi R., Haygood M.G., Tebo B.M. (1996). Unusual Ribulose-1,5-Bisphosphate Carboxylase/Oxygenase Genes from a Marine Manganese-Oxidizing Bacterium. Microbiology.

[11] Ercole C., Altieri F., Piccone C. (1999). Influence of Manganese Dioxide and Manganic Ions on the Production of Two Proteins in Arthrobacter Sp. Geomicrobiol. J..

[12] Lovley D.R., Chapelle F.H. (1995). Deep Subsurface Microbial Processes. Rev. Geophys..

[13] Trimble R.B., Ehrlich H.L. (1968). Bacteriology of Manganese Nodules. Appl. Microbiol..

[14] Sedlakova-Kadukova J. (2022). Microorganisms in Metal Recovery—Tools or Teachers. Microbial Syntrophy-Mediated Eco-Enterprising.

[15] Johnson D.B. (2014). Biomining—Biotechnologies for Extracting and Recovering Metals from Ores and Waste Materials. Curr. Opin. Biotechnol..

[16] Acharya C., Kar R.N., Sukla L.B. (2003). Studies on Reaction Mechanism of Bioleaching of Manganese Ore. Miner. Eng..

[17] Nkuna R., Ijoma G.N., Matambo T.S., Chimwani N. (2022). Accessing Metals from Low-Grade Ores and the Environmental Impact Considerations: A Review of the Perspectives of Conventional versus Bioleaching Strategies. Minerals.

[18] Mizrahi-Man O., Davenport E.R., Gilad Y. (2013). Taxonomic Classification of Bacterial 16S RRNA Genes Using Short Sequencing Reads: Evaluation of Effective Study Designs. PLoS ONE.

[19] Bolyen E., Rideout J.R., Dillon M.R., Bokulich N.A., Abnet C.C., Al-Ghalith G.A., Alexander H., Alm E.J., Arumugam M., Asnicar F. (2019). Reproducible, Interactive, Scalable and Extensible Microbiome Data Science Using QIIME 2. Nat. Biotechnol..

[20] Bernardini S., Bellatreccia F., Columbu A., Vaccarelli I., Pellegrini M., Jurado V., del Gallo M., Saiz-Jimenez C., Sodo A., Millo C. (2021). Morpho-Mineralogical and Bio-Geochemical Description of Cave Manganese Stromatolite-Like Patinas (Grotta Del Cervo, Central Italy) and Hints on Their Paleohydrological-Driven Genesis. Front. Earth Sci..

[21] Cappuccino J.G., Welsh C.T., Cappuccino J.G., Welsh C.T. (2017). Microbiology: A Laboratory Manual, Global Edition.

[22] Stamatakis A., Ludwig T., Meier H. (2005). RAxML-III: A Fast Program for Maximum Likelihood-Based Inference of Large Phylogenetic Trees. Bioinformatics.

[23] Bauer M.A., Kainz K., Carmona-Gutierrez D., Madeo F. (2018). Microbial Wars: Competition in Ecological Niches and within the Microbiome. Microb. Cell.

[24] Akob D.M., Bohu T., Beyer A., Schäffner F., Händel M., Johnson C.A., Merten D., Büchel G., Totsche K.U., Küsel K. (2014). Identification of Mn(II)-Oxidizing Bacteria from a Low-PH Contaminated Former Uranium Mine. Appl. Environ. Microbiol..

[25] Hou D., Zhang P., Wei D., Zhang J., Yan B., Cao L., Zhou Y., Luo L. (2020). Simultaneous Removal of Iron and Manganese from Acid Mine Drainage by Acclimated Bacteria. J. Hazard. Mater..

[26] Yang H., Tang X., Luo X., Li G., Liang H., Snyder S. (2021). Oxidants-Assisted Sand Filter to Enhance the Simultaneous Removals of Manganese, Iron and Ammonia from Groundwater: Formation of Active MnOx and Involved Mechanisms. J. Hazard. Mater..

[27] Wang Y., Tsang Y.F., Wang H., Sun Y., Song Y., Pan X., Luo S. (2020). Effective Stabilization of Arsenic in Contaminated Soils with Biogenic Manganese Oxide (BMO) Materials. Environ. Pollut..

[28] Volokhin Y.G., Mikhailik P.E., Mikhailik E.V. (2020). Minerals in Manganese Deposits of Belyaevsky Volcano, the Sea of Japan. Russ. J. Pac. Geol..

[29] Carmichael I.S.E. (1967). The Mineralogy and Petrology of the Volcanic Rocks from the Leucite Hills, Wyoming. Contrib. Mineral. Petrol..

[30] Singer A., Zarei M., Lange F.M., Stahr K. (2004). Halloysite Characteristics and Formation in the Northern Golan Heights. Geoderma.

[31] Barile M. (2010). Caratterizzazione Funzionale Della Comunita’ Macrobentonica Dei Fiumi Della Tuscia e Analisi Delle Relazioni Con Le Variabili Abiotiche a Diversa Scala Spaziale. Ph.D. Thesis.

[32] Beal E.J., House C.H., Orphan V.J. (2009). Manganese- and Iron-Dependent Marine Methane Oxidation. Science.

[33] Rajabzadeh M.A., Haddad F., Polgári M., Fintor K., Walter H., Molnár Z., Gyollai I. (2017). Investigation on the Role of Microorganisms in Manganese Mineralization from Abadeh-Tashk Area, Fars Province, Southwestern Iran by Using Petrographic and Geochemical Data. Ore Geol. Rev..

[34] Yu W., Polgári M., Gyollai I., Fintor K., Szabó M., Kovács I., Fekete J., Du Y., Zhou Q. (2019). Microbial Metallogenesis of Cryogenian Manganese Ore Deposits in South China. Precambrian Res..

[35] Ijaz A., Mumtaz M.Z., Wang X., Ahmad M., Saqib M., Maqbool H., Zaheer A., Wang W., Mustafa A. (2021). Insights into Manganese Solubilizing Bacillus Spp. for Improving Plant Growth and Manganese Uptake in Maize. Front. Plant Sci..

[36] Sanket A.S., Ghosh S., Sahoo R., Nayak S., Das A.P. (2017). Molecular Identification of Acidophilic Manganese (Mn)-oxide solubilizing Bacteria from Mining Effluents and Their Application in Mineral Beneficiation. Geomicrobiol. J..

[37] Kanso S., Greene A.C., Patel B.K.C. (2002). Bacillus Subterraneus Sp. Nov., an Iron- and Manganese-Reducing Bacterium from a Deep Subsurface Australian Thermal Aquifer. Int. J. Syst. Evol. Microbiol..

[38] Sujith P.P., Mourya B.S., Krishnamurthi S., Meena R.M., Loka Bharathi P.A. (2014). Mobilization of Manganese by Basalt Associated Mn(II)-Oxidizing Bacteria from the Indian Ridge System. Chemosphere.

[39] Gupta R.S., Patel S., Saini N., Chen S. (2020). Robust Demarcation of 17 Distinct Bacillus Species Clades, Proposed as Novel Bacillaceae Genera, by Phylogenomics and Comparative Genomic Analyses: Description of Robertmurraya Kyonggiensis Sp. Nov. and Proposal for an Emended Genus Bacillus Limiting It only to the members of the Subtilis and Cereus clades of species. Int. J. Syst. Evol. Microbiol..

[40] Schmid J., Auling G. (1987). Manganese Transport in Brevibacterium Ammoniagenes ATCC *J*. Bacteriol..

[41] Willing A., Follmann H., Auling G. (1988). Ribonucleotide Reductase of Brevibacterium Ammoniagenes Is a Manganese Enzyme. Eur. J. Biochem..

[42] Gillard B., Chatzievangelou D., Thomsen L., Ullrich M.S. (2019). Heavy-Metal-Resistant Microorganisms in Deep-Sea Sediments Disturbed by Mining Activity: An Application Toward the Development of Experimental in Vitro Systems. Front. Mar. Sci..

[43] Shulse C.N., Maillot B., Smith C.R., Church M.J. (2017). Polymetallic Nodules, Sediments, and Deep Waters in the Equatorial North Pacific Exhibit Highly Diverse and Distinct Bacterial, Archaeal, and Microeukaryotic Communities. Microbiologyopen.

[44] Lindh M.v., Maillot B.M., Shulse C.N., Gooday A.J., Amon D.J., Smith C.R., Church M.J. (2017). From the Surface to the Deep-Sea: Bacterial Distributions across Polymetallic Nodule Fields in the Clarion-Clipperton Zone of the Pacific Ocean. Front. Microbiol..

[45] Gillan D.C., Baeyens W., Bechara R., Billon G., Denis K., Grosjean P., Leermakers M., Lesven L., Pede A., Sabbe K. (2012). Links between Bacterial Communities in Marine Sediments and Trace Metal Geochemistry as Measured by in Situ DET/DGT Approaches. Mar. Pollut. Bull..

[46] Walsh E.A., Kirkpatrick J.B., Pockalny R., Sauvage J., Spivack A.J., Murray R.W., Sogin M.L., D’Hondt S. (2016). Relationship of Bacterial Richness to Organic Degradation Rate and Sediment Age in Subseafloor Sediment. Appl. Environ. Microbiol..

[47] Singh R.L., Singh P.K. (2017). Global Environmental Problems. Principles and Applications of Environmental Biotechnology for a Sustainable Future.

[48] Das A.P., Ghosh S., Mohanty S., Sukla L.B., Sukla L.B., Pradhan N., Panda S., Mishra K.B. (2015). Advances in Manganese Pollution and Its Bioremediation. Environmental Microbial Biotechnology.

[49] Mourinha C., Palma P., Alexandre C., Cruz N., Rodrigues S.M., Alvarenga P. (2022). Potentially Toxic Elements’ Contamination of Soils Affected by Mining Activities in the Portuguese Sector of the Iberian Pyrite Belt and Optional Remediation Actions: A Review. Environments.

