# Supplementary material for: The “Infernaccio” Gorges: Microbial Diversity of Black Deposits and Isolation of Manganese-Solubilizing Bacteria
Source: Biology (Basel). 2022 Aug 11;11(8):1204. doi: 10.3390/biology11081204 (PMC9404752; doi:10.3390/biology11081204)
Supplement: Supplementary file 1 [file biology-11-01204-s001.zip › biology-1796459-supplementary.pdf]

Supplementary Material

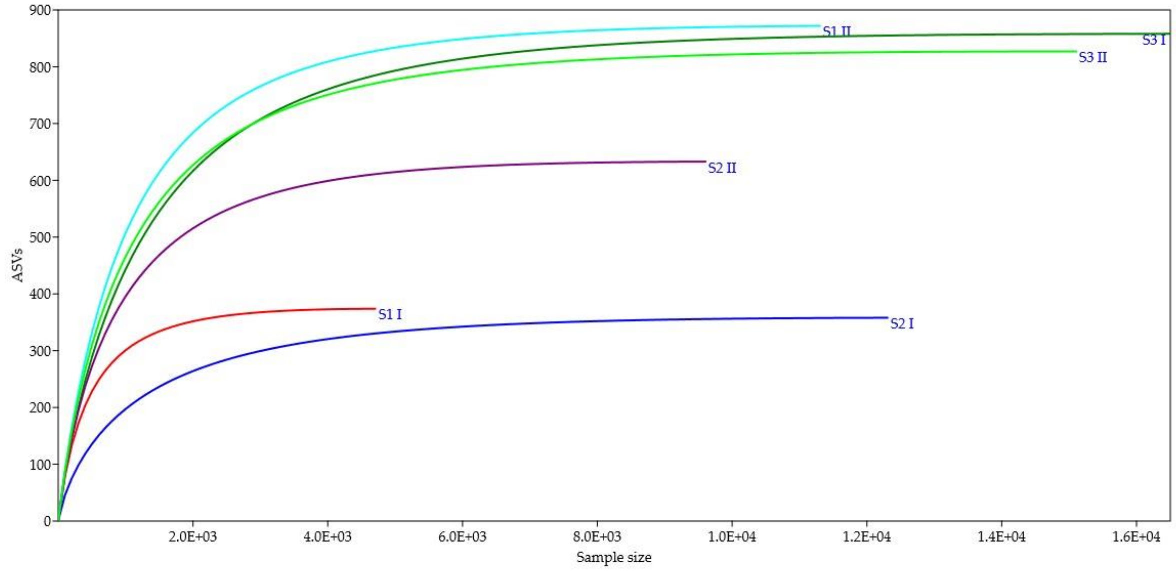

Figure S1. Species accumulation plot showing the number of amplicon sequence variants (ASVs) on sample size for each sample.

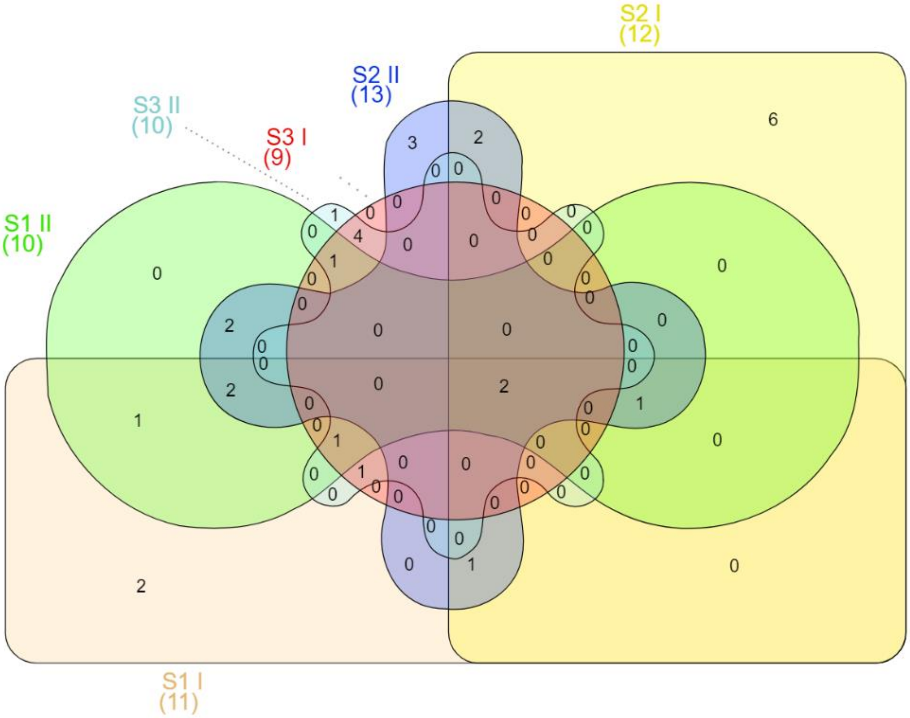

Figure S2. Venn diagram underlining the common ASVs (give in brackets) at the family level. In the Figure [S1 I]: env.OPS 17,Xanthomonadaceae; [S2 I]: Bdellovibrionaceae, Hyphomonadaceae, Ilumatobacteraceae, Rhodanobacteraceae, Rhodobacteraceae, Sporichthyaceae; [S3 II]: Pyrinomonadaceae; [S2 II]: Methylomonaceae, Methylophilaceae, Opitutaceae; [S3 I] and [S3 II]: Chthoniobacteraceae, Nocardioidaceae, Solirubrobacteraceae, Xanthobacteraceae; [S1 I] and [S1 II]: Flavobacteriaceae; [S1 II] and [S2 II]: Pedosphaeraceae, Solibacteraceae (Subgroup 3); [S1 I] and [S2 I] and [S2 II]: Burkholderiaceae; [S1 I] and [S1 II] and [S2 II]: Microscillaceae, Nitrosomonadaceae; [S1 I] and [S2 I] and [S1 II] and [S2 II]: Nitrospiraceae; [S1 I] and [S3 I] and [S1 II] and [S3 II]: Gemmatimonadaceae; [S1 I] and [S3 I] and [S3 II]: Sphingomonadaceae; [S1 I] and [S2 I] and [S3 I] and [S1 II] and [S2 II] and [S3 II]: uncultured, unknown; [S3 I] and [S1 II] and [S3 II]: TRA3-20; [S2 I] and [S2 II]: Chitinophagaceae, Gallionellaceae.

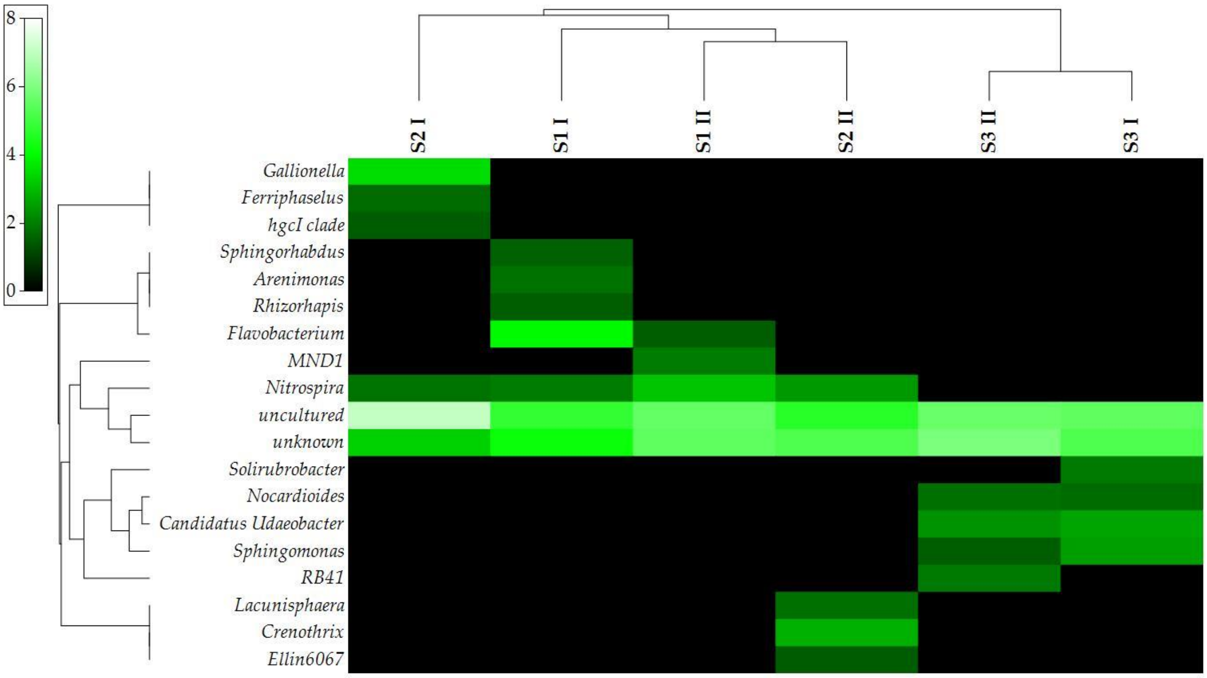

Figure S3. Heatmap of the main genera found in the samples. The upper clusterization shows the sample distribution according to taxa presence/absence and abundances. The heat map was realized based on a resemblance matrix (Analysis between variables and measure of the index of association) and hierarchical cluster analysis (Group average cluster mode).

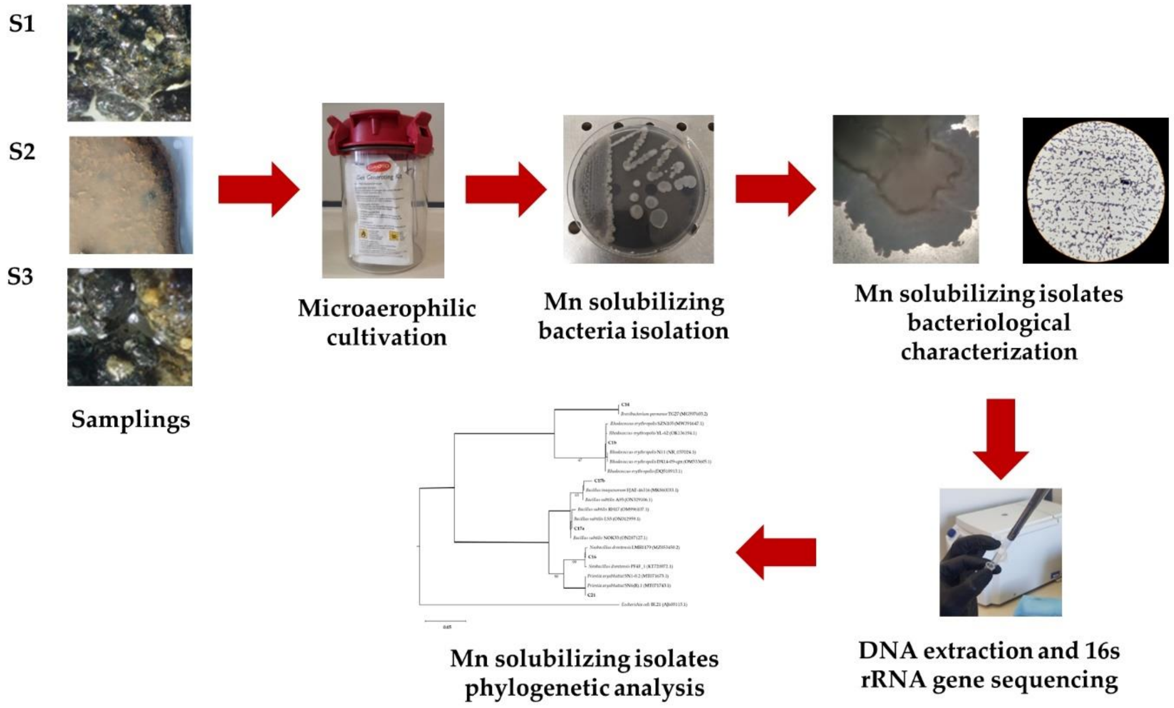

Figure S4. Summary of the experiment setup during Mn-oxide solubilizing process.

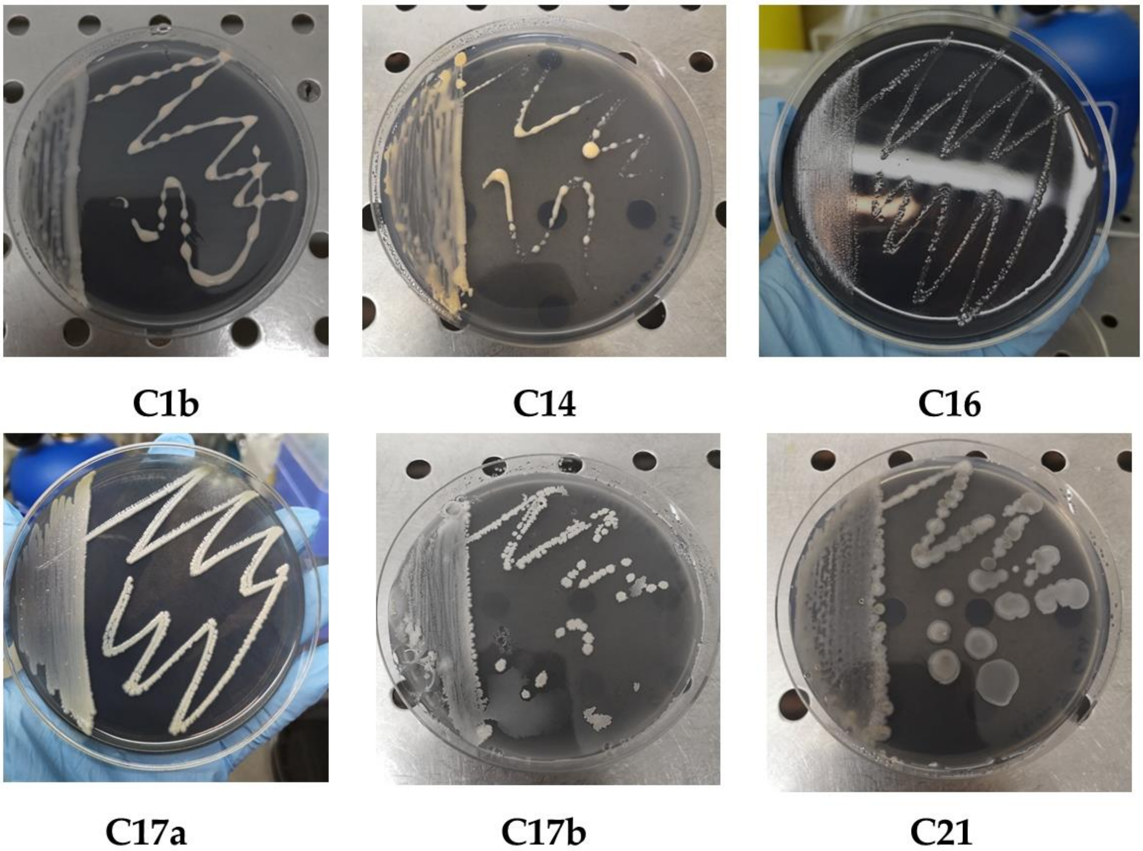

Figure S5. Mn-oxide solubilizing isolates on manganese basal medium (MMB).
